# Epidemiology of diabetes

**DOI:** 10.1016/j.mpmed.2014.09.007

**Published:** 2014-12

**Authors:** Nita Gandhi Forouhi, Nicholas J. Wareham

**Affiliations:** **Nita Gandhi Forouhi MBBS BMedSci PhD MRCP FFPHM** is Programme Leader and Honorary Public Health Physician at the MRC Epidemiology Unit, University of Cambridge, UK. Competing interests: none declared; **Nicholas J Wareham MBBS PhD FRCP FFPHM** is Director of the MRC Epidemiology Unit, University of Cambridge and Honorary Consultant, Addenbrooke's Hospital, Cambridge, UK. Competing interests: none declared

**Keywords:** Aetiology, diagnosis, epidemiology, prevention, screening, type 1 diabetes, type 2 diabetes

## Abstract

The disease burden related to diabetes is high and rising in every country, fuelled by the global rise in the prevalence of obesity and unhealthy lifestyles. The latest estimates show a global prevalence of 382 million people with diabetes in 2013, expected to rise to 592 million by 2035. The aetiological classification of diabetes has now been widely accepted. Type 1 and type 2 diabetes are the two main types, with type 2 diabetes accounting for the majority (>85%) of total diabetes prevalence. Both forms of diabetes can lead to multisystem complications of microvascular endpoints, including retinopathy, nephropathy and neuropathy, and macrovascular endpoints including ischaemic heart disease, stroke and peripheral vascular disease. The premature morbidity, mortality, reduced life expectancy and financial and other costs of diabetes make it an important public health condition.

## Type 1 diabetes

The acute onset of type 1 diabetes mellitus and its rapid presentation to medical attention facilitates accurate registering of new cases. Provided ascertainment can be verified, these data can be combined with population denominator data to give age-specific and sex-specific incidences.

### Geographical variation

**Figure 1:**
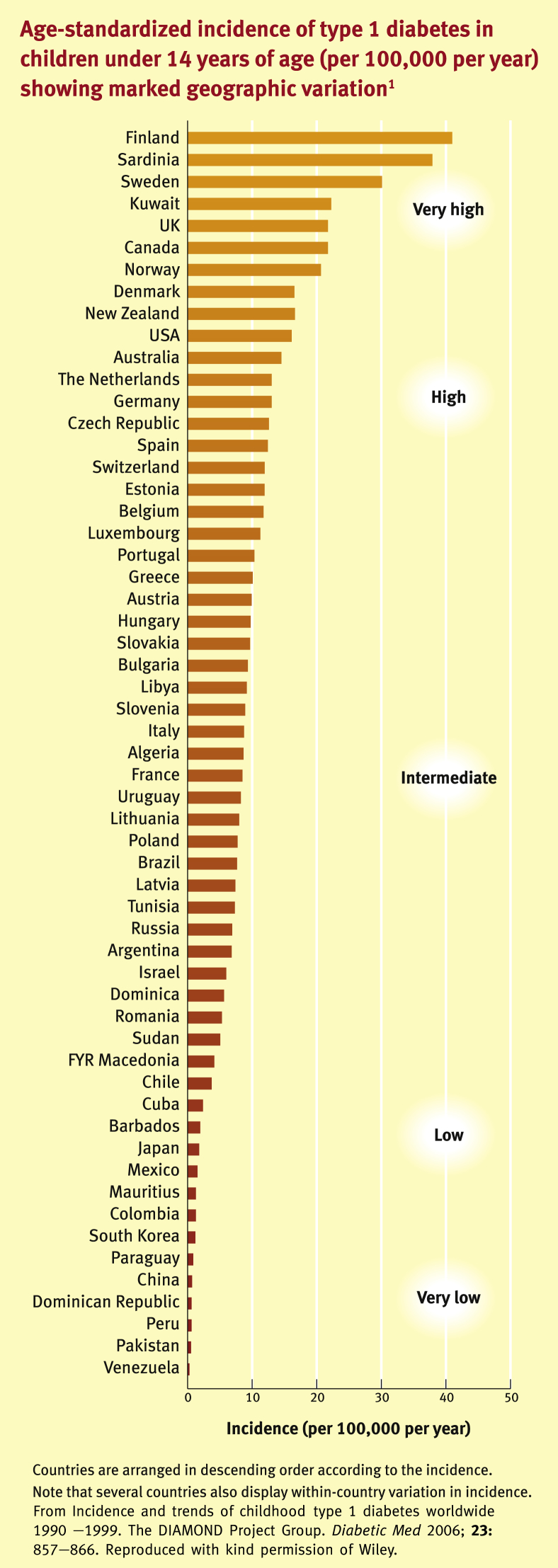


The incidence of type 1 diabetes in children varies nearly 400-fold between countries (Figure 1) with age-adjusted incidence rates ranging from 0.1 per 100,000 per year in parts of Venezuela and China to 37.8 in Sardinia and 40.9 per 100,000/year in Finland.^1^ The high rate observed in Sardinia is notably discordant with the incidence in Italy as a whole. Incidence also varies within several other countries including China, where there is a 12-fold variation by region (0.13–1.61/100,000). In general, countries in Europe and North America have either high or intermediate incidences. The incidence in Africa is generally intermediate and that in Asia is low, with the notable exception of Kuwait.

### Variation with age, sex, and ethnicity

Type 1 diabetes can occur at any age, but in most populations the incidence is highest between birth and 14 years old. Generally, all populations display a steady increase in incidence rate with age up to around 10–15 years. There are no population-based incidence data for ages above 35 years. Overall, there is a slight male excess among children in high-incidence countries, while the opposite is seen in low-incidence countries, but these differences are small.^1^ However, there is generally a male excess among young adults,^2^ and peak incidence is around puberty in most populations. Mirroring the geographic pattern, incidence is higher in populations of European origin than in non-Europeans.^1^

### Temporal variation

Average increases of 2.8–3.0% per year worldwide^1^ and of 3.9% per year in Europe ^3^ have been reported. Generally, the relative magnitude of increase is greater in low-incidence countries. The most pronounced increase is in the youngest age group (0–4 years).^1,3^ The incidence of type 1 diabetes also varies with season, being highest in autumn and winter.^4^

### Aetiological factors

Genetic susceptibility is important but not sufficient in causation of type 1 diabetes. Environmental factors have a more important role in progression from islet autoimmunity to overt disease, possibly because improved living standards have reduced microorganism exposure, leading to increased autoimmunity. Case-control studies have shown associations with early social mixing, viral infections, toxins and dietary factors such as exclusive breast-feeding and delayed introduction of cows' milk. Hypothesized aetiological factors include a vitamin D deficiency,^5^ which has been implicated by some genetics studies.^6^ Suggestions for a role of omega-3 fatty acids have also been made.^7^

## Type 2 diabetes

The slow onset of type 2 diabetes, and its usual presentation without the acute metabolic disturbance seen in type 1 diabetes, means that the true time of onset is difficult to determine. There is also a long pre-detection period, and up to one-half of cases in the population may be undiagnosed.

### Diagnosis of type 2 diabetes

Because the ratio of detected to undetected cases may vary over time and between places, epidemiological research aimed at defining the true prevalence of type 2 diabetes has relied on special studies in which the presence and absence of disease are defined by the oral glucose tolerance test (OGTT). The WHO recommends use of the 75 g OGTT, with diabetes defined by fasting glucose 7.0 mmol/L or more and/or 2-hour post-challenge glucose 11.1 mmol/L or more. Recently, the American Diabetes Association, the WHO and other authoritative bodies have approved the use of HbA_1c_ for the diagnosis of diabetes with a cut-off of 48 mmol/mol (>6.5%) in a standardized laboratory.

### Variation in prevalence by geographical location, ethnicity, age, and sex

**Figure 2:**
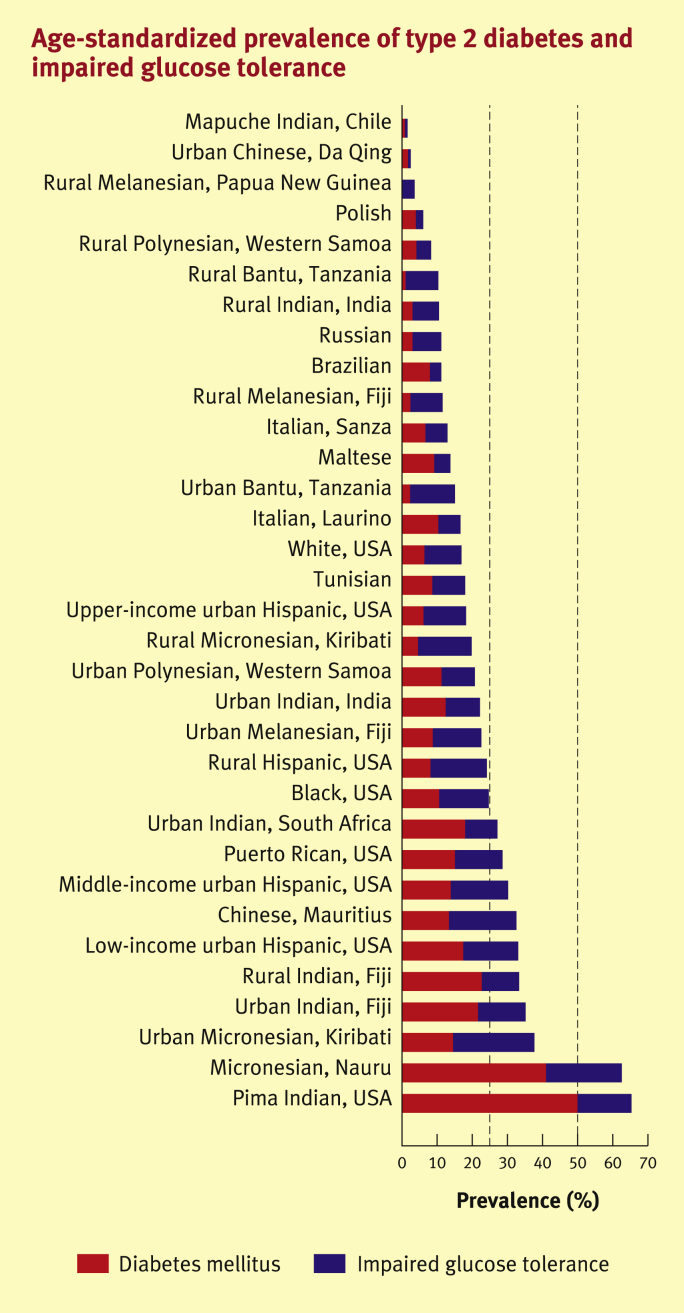
Age-standardized prevalence of type 2 diabetes and impaired glucose tolerance.

Figure 2 shows the age-standardized prevalence of type 2 diabetes and impaired glucose tolerance (IGT). As in type 1 diabetes, there is marked geographical variation, but the pattern is different. The prevalence is lowest in rural areas of developing countries, generally intermediate in developed countries, and highest in certain ethnic groups, particularly those that have adopted Western lifestyle patterns. Populations with the highest prevalence have a high prevalence of obesity. It is hypothesized that genetic susceptibility to obesity would be disadvantageous in times of food abundance, but advantageous when food is scarce, driving its persistence by natural selection. This ‘thrifty genotype’ hypothesis is supported by evidence of gene–environment interaction; individuals who migrate from low-prevalence areas to the West are at increased risk of type 2 diabetes. Diabetes is up to fourfold to sixfold more prevalent in South Asians and African-Caribbeans in the UK compared with European white populations.^8^ There is only a small gender difference in the global numbers of people with diabetes, with about 14 million more men than women estimated to have diabetes in 2013.^9^ The prevalence increases sharply with age in both sexes.^9^

### Incidence and temporal variation

From studies using serial glucose tolerance testing the annual incidence in Europeans is around 7 per 1000 per year in Western populations.^10^ The incidence in individuals known to have IGT is about tenfold greater than in those with normal glucose tolerance.^11^ The risk of future progression to diabetes is also greater in those with other hyperglycaemic states, including gestational diabetes mellitus. Data from the US Centre for Disease Control show a near quadrupling of diagnosed diabetes from 5.5 million persons in 1980 to 21.1 million in 2010. This increase mirrors the increasing prevalence of obesity. Worldwide, there is a projected increase in the prevalence of diabetes from 382 million (8.3%) in 2013 to 592 million (10.1%) in 2035.^9^ Estimates of the prevalence in developing countries show even more marked increases, particularly in areas where populations are rapidly adopting Western lifestyles (Table 1).^9^ The increase in the prevalence of obesity in childhood has led to the appearance of type 2 diabetes in children and young adults, particularly those in highly susceptible ethnic groups. In the United States, prevalence of type 2 diabetes in youth (age 10–19 years) was higher in American Indian, black and Hispanic youth compared with white youth, and increased overall from 0.34 per 1000 (95% confidence interval 0.31–0.37) in 2001 to 0.46 per 1000 (95% CI, 0.43–0.49) in 2009.^12^

### Aetiological factors

Prospective studies suggest that the main pathophysiological defects leading to type 2 diabetes are insulin resistance and a relative insulin secretory defect. The main aetiological risk factors are age, obesity, family history, and physical inactivity. Dietary risk factors have recently emerged: risk is increased by high consumption of red and processed meat^13^ and sugar-sweetened beverages,^14^ and reduced by intake of fruit and vegetables,^15^ some types of dairy products,^16^ and some overall dietary patterns.^17^ Novel strategies to use quantifiable nutritional biomarkers are paving the way for more detailed understanding of the association between diet and diabetes. Although the heritability of type 2 diabetes is high (30–70%) and more than 60 genetic variants related with diabetes risk have now been identified,^18^ the individual effects of genetic variants are modest, and even when combined into a genetic score, known genes contribute little to the prediction of diabetes. Phenotype-based risk models provide greater discrimination for diabetes, and the addition of genotypic information adds no more than 5–10% improvement in prediction. The current conclusion is that genetic variants provide insights into biological pathways and pathogenesis of diabetes, but not its prediction. It is likely that interactions between the environment/lifestyle and genetic factors provide the explanation for the risk of type 2 diabetes, but demonstrating such interaction is challenging. Encouraging research findings have recently shown higher absolute risk of diabetes associated with obesity at any level of genetic risk.^19^

## Prevention and screening

### Primary prevention

Randomized clinical trials in several countries have provided evidence that, in high-risk individuals with IGT, progression to type 2 diabetes can be reduced by intensive lifestyle intervention with diet or physical activity, or with drug therapy using glucose-lowering agents such as metformin.^20^ In addition to their clinical effectiveness, there is now also evidence for the cost effectiveness of these interventions. The challenges that remain are to determine how high-risk individuals should be identified, and how lifestyle changes of healthier diet and regular physical activity can be sustained The evidence from randomized controlled trials that diabetes can be prevented does not prove that intervention in high-risk individuals is the most appropriate strategy for prevention in real world settings. As in many areas of primary prevention, high-risk approaches can be effective for the individuals included in the programmes but have a limited impact on the public health burden of diabetes. Complementary approaches that seek to make small shifts in the population distribution of dietary and physical activity behaviours are required. Such approaches make relatively little difference in risk at the individual level but have a major impact on the public health burden of diabetes when that risk reduction is summated across large numbers of people in the population.^21^ The future challenge involves finding ways of integrating high-risk and population approaches to prevention, and balancing relative investment in the two strategies.

### Secondary prevention: screening

As it is estimated that the onset of type 2 diabetes occurs an average of about 4–7 years before clinical diagnosis, and as a high proportion of individuals exhibit evidence of end-organ damage by that point, screening has been proposed in the hope that early detection and early treatment would reduce long-term burden. However there is no definitive evidence that screening results in net benefit and most authorities have proposed opportunistic rather than systematic screening targeting high-risk sub-groups. Recent data have emerged from the ADDITION-Europe, a pragmatic primary care-based trial of intensive multifactorial treatment compared with routine care on cardiovascular outcomes among individuals with screen-detected diabetes. This trial shows that:

• Screening for diabetes is feasible, and has limited short- and long-term adverse psychological impact.
• Cardiovascular risk factors (blood pressure, cholesterol and weight), including lifestyle behaviours (smoking), improve markedly following screen detection of diabetes, even among those receiving routine general practice care.
• At the population level, invitation of high-risk individuals to screening is not associated with a reduction in all-cause or diabetes-related mortality over 10 years.^22,23^

Uncertainties remain concerning optimal strategies to increase uptake of screening, to deliver care to screen-detected patients and to manage those who screen negative but are at high risk of diabetes and cardiovascular disease, as well as the overall cost-effectiveness of screening programmes. Rather than screening whole populations for diabetes, primary care teams should focus efforts on earlier detection, lifestyle advice and intensive treatment of risk factors among individuals at high risk of diabetes and cardiovascular disease.

## Acknowledgement

NGF and NJW acknowledge support from the core Medical Research Council Epidemiology Unit Programmes (MC_UU_12015/5 and MC_UU_12015/1).

## Figures and Tables

**Table 1:** Top ten countries for number of people aged 20–79 years with diabetes in 2013 and 2035 (a) and top ten countries for diabetes prevalence (%) in 2013 and 2035 (b)

	2013		2035	
	Country	No. of adults with diabetes (millions)	Country	No. of adults with diabetes (millions)
**a**				
1	China	98.4	China	142.7
2	India	65.1	India	109.0
3	USA	24.4	USA	29.7
4	Brazil	11.9	Brazil	19.2
5	Russian Federation	10.9	Mexico	15.7
6	Mexico	8.7	Indonesia	14.1
7	Indonesia	8.5	Egypt	13.1
8	Germany	7.6	Pakistan	12.8
9	Egypt	7.5	Turkey	11.8
10	Japan	7.2	Russian Federation	11.2

	2013		2035	
	Country	Prevalence (%)	Country	Prevalence (%)
**b**				
1	Tokelau	37.5	Tokelau	37.9
2	Federated States of Micronesia	35.0	Federated States of Micronesia	35.1
3	Marshall Islands	34.9	Marshall Islands	35.0
4	Kiribati	28.8	Kiribati	28.9
5	Cook Islands	25.7	Cook Islands	25.7
6	Vanuatu	24.0	Saudi Arabia	24.5
7	Saudi Arabia	24.0	Vanuatu	24.2
8	Nauru	23.3	Nauru	23.3
9	Kuwait	23.1	Kuwait	23.2
10	Qatar	22.9	Qatar	22.8
